# Retracted: Extra Dose of Vitamin C Based on a Daily Supplementation Shortens the Common Cold: A Meta-Analysis of 9 Randomized Controlled Trials

**DOI:** 10.1155/2023/9848057

**Published:** 2023-04-10

**Authors:** BioMed Research International

*BioMed Research International* and the authors have retracted the article titled “Extra Dose of Vitamin C Based on a Daily Supplementation Shortens the Common Cold: A Meta-Analysis of 9 Randomized Controlled Trials” [1] due to a fundamental error identified in the calculations underlying the conclusions, as raised by readers of the article, Dr. Colby Vorland and Dr. Andrew Brown of the Indiana University School of Public Health-Bloomington, and Dr. Harri Hemilä of the University of Helsinki, summarized on PubPeer: https://pubpeer.com/publications/077EAB95E9141BE6A455C30F0A688E.

The meta-analysis contains multiple instances of an error in which the placebo group has been double-counted in trials with more than two intervention arms, for example, in Figures 2-10. When the calculations are corrected, effect size estimates and confidence intervals change, with *p*-values in at least some cases becoming not statistically significant at a designation of 0.05. The authors and the journal are continuing to investigate the impact of the errors on the conclusions, and a revised version of the article may be considered for publication should the issues be resolved.

Given the potential extent of the impact of this error on the conclusions, the Editorial Board recommends the retraction of the article with the agreement of the authors.
